# Syrian medical, dental and pharmaceutical publication in the last decade: A bibliometric analysis

**DOI:** 10.1016/j.amsu.2021.102441

**Published:** 2021-05-26

**Authors:** Muhamad Zakaria Brimo Alsaman, Hala Sallah, Rayan Badawi, Anas Ghawi, Mohammad Nour Shashaa, Luma Haj Kassem, Ahmad Ghazal

**Affiliations:** a Faculty of Medicine, University of Aleppo, Aleppo, Syria; b Department of Otolaryngology, Faculty of Medicine, University of Aleppo, Aleppo, Syria; c Department of Surgery, Aleppo University Hospital, Aleppo, Syria; d CME Office, Faculty of Medicine, University of Aleppo, Aleppo, Syria

**Keywords:** Bibliometric analysis, Syrian crisis, Medicine, Dentistry, Pharmacy, EBM, Evidence-Based-Medicine, CME, Continuous Medical Education

## Abstract

**Background:**

Scientific research has an essential role in building up the basics of public health and clinical care. As a developing country, Syria has a limited contribution to scientific research. Besides, the current Syrian crisis has inflicted severe damage to the infrastructure of the country, which, in turn, negatively affected the scientific sphere. This study examines the scientific publishing in Syria from 2011 until 2020. It aims to define the real and deep reasons for the slow-down in publication to get over them, push Syria to keep track of the latest updates, and take its place in scientific research.

**Methods:**

We conducted a bibliometric analysis of articles published in (PubMed and Scopus) Databases from 1/1/2011 until 26/12/2020, using the following search terms ((“Syrian Arab Republic”) OR (Syria) OR (Syrian)) limiting the search to (Affiliation) fields.

**Results:**

Syrian medical, dental and pharmaceutical institutions published 1403 papers from 2011 until 2020. There were only 55 papers in 2011, and a peak with 180 papers in 2018. Besides, publications in the last 4 years were 1.135 times more than publications in the last 6 years.

**Conclusion:**

We noticed a peak in quantity of Syrian medical, dental and pharmaceutical publications in the last decade**.** Accordingly, we recommend enhancing research skills, paying more attention to the quality of researches, and holding research workshops and Evidence Based conferences to enhance the scientific endeavor.

## Highlights

• We conducted a novel bibliometric analysis to evaluate the research productivity in the current crisis.
• Our Results show that papers in Medical, Dental and Pharmaceutical field have been increasing every year.
• Otherwise, there is a paucity in publishing compared with equivalent countries.
• It takes Syria as an example of poor countries' research interest so it may inspire those have similar circumstances.

## 1 Background

Public health is limitless, especially with continuous sharing of researches and evidences worldwide. Publications and researches have an essential role in clinical care, teaching, and training [1].

Nowadays, the scientific field is witnessing a massive acceleration in publishing, with increased quality and quantity of published papers. This can be explained by the well-known role of scientific research in expanding knowledge, supporting job progression, and providing professional education as a form of continuous medical education (CME) [1,2].

There are many acceptable methods for evaluating the scientific level of individuals and countries. One of them – which was published by South Korean plastic surgeons-considers the number of published papers as an evidence of quantitative evaluation of researches [3]. Therefore, publishing has become an indicator of the scientific development of a country.

In recent years, Syria become the spot of attention after the devastating war has caused severe damage to the infrastructures, and serious threats to the functioning of educational, economic, and social institutions [4]. Even before the war, as a developing country, Syria had a minimal participation in global research. Diab et al. noted that even though the Syrian medical research productivity from 1988 to 2011 increased, the medical and scientific society has to exert more efforts to improve both quality and quantity of published papers [5].

Despite obstacles and challenges that faced the research process, Syrian students-medical students in particular-are attempting to keep up with the latest medical research findings and EBM [6].

We conducted a novel bibliometric analysis to evaluate the research productivity in the current crisis which takes a comprehensive look of the scientific publishing in Syria from 2011 until 2020. It aims to define the real reasons behind the current situation in order to get over them, push Syria to keep looking for the latest updates, and take place in scientific research as a major contributor.

## 2 Methods

### 2.1 Strategy of search

In this study, we conducted a systematic electronic database search for relevant studies from 1/1/2011 until 26/12/2020 in PubMed and Scopus data bases, using the following search terms: ((“Syrian Arab Republic”) OR (Syria) OR (Syrian)) limiting the search to (Affiliation) field.

### 2.2 Inclusion and exclusion criteria

We included papers that at least one of their authors was affiliated to one of Syrian medical, dental, or pharmaceutical institutions (Damascus, Aleppo, Latakia, Hama, Homs, Tartous and Daraa and their affiliated institutions).

We excluded all other fields and articles that their authors did not affiliate to medical, dental, or pharmaceutical institutions. There were no restrictions on study design or language.

### 2.3 Data collection and statistical analysis

One author (MZBA) developed the data extraction using Endnote X8 and formatted it using Microsoft Excel. The extracted data included year, date, title, abstract, and authors' names and their affiliations. We removed duplicated data for the combined database, and six authors (MZBA, HS, RB, AG, MNS, LHK) scanned the papers according to our inclusion and exclusion criteria to select appropriate articles. We included 1403 articles for bibliometric analysis, and manually defined: city (Damascus, Aleppo, Latakia, Hama, Homs, Tartous and Daraa), field (medicine, dentistry and pharmacy) depending on article's field, research type (basic scientific research: in vitro or on animals, primary observational or interventional research, and secondary research) and specialties for medical articles. Every marked phase was done twice by two different authors.

We did descriptive statistics (number of publication and percentage) using Microsoft Excel 2019 to present our Results in tables and figures.

## 3 Results

**Fig. 1:**
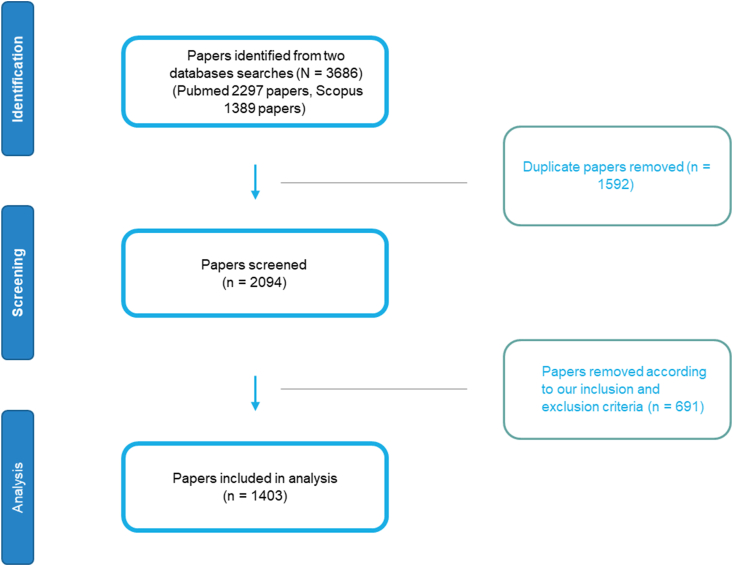
Flow chart of the search Results and articles selection process.

From initial search on two databases depending on our search terms, we found 3686 papers, and after removing the duplicated studies and manual search exclusions, we ended up with only 1403 papers that met our inclusion criteria and then we included them in the analysis (Fig. 1).

We found that Syrian medical, dental and pharmaceutical institutions published 1403 papers in total, in the period from 2011 until 26/12/2020.

The number of published papers has been increasing by years. It started in 2011 with only 55 published papers and then reached 279 in 26/12/2020 (Table 1).

**Table 1:** Number of research papers in the last decade.

Year	Number of publications	Frequency
2011	55	3.9%
2012	89	6.3%
2013	102	7.3%
2014	108	7.7%
2015	130	9.3%
2016	130	9.3%
2017	137	9.8%
2018	180	12.8%
2019	193	13.8%
2020	279	19.9%
	1403	100%

**Fig. 2:**
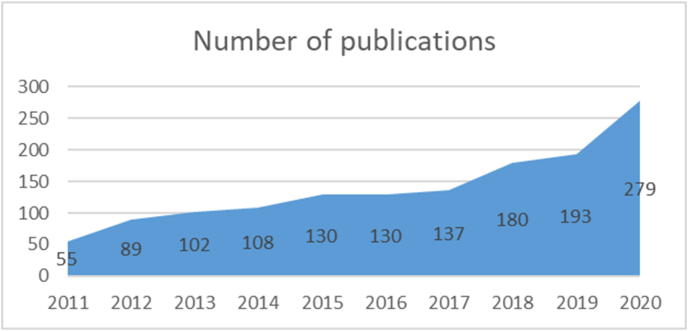
Frequency polygon shows the increase in the number of research papers in the last decade.

The significant peak was in 2018 with 180 papers. We noticed that the last 4 years' publications counted 1.135 times more than the last 6 years' publications (Fig. 2).

**Fig. 3:**
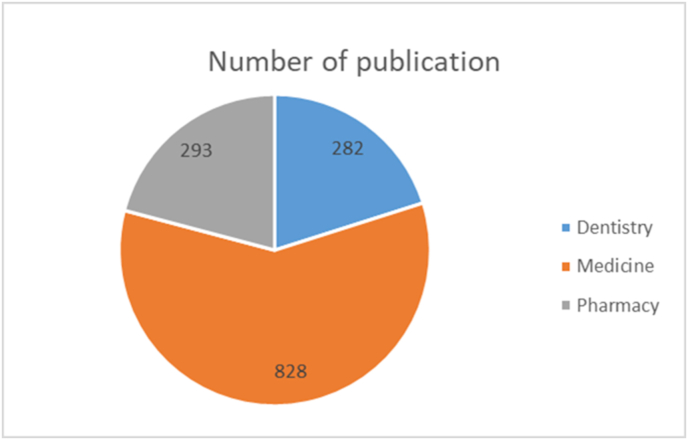
Bar chart illustrates the frequency of papers in medicine, dentistry and pharmacy.

Out of 1403 papers; 828 (59.0%) were in the medical field, while 282 (20.1%) were in dentistry, and 293 (20.9%) papers were in pharmacy (Fig. 3).

Damascus University and its affiliated institutions published 58% of total papers, which is the highest number of publications with 863 papers. Aleppo University and its affiliated institutions came next with 349 papers, while Latakia University and its affiliated institutions took the third place with 132 papers. Hama, Homs and their affiliated institutions participated in 34 and 31 papers, respectively. Dara'a University and its affiliated institutions took the last place with only 19 papers (Table 2).

**Table 2:** Number of publications were produced by an individual institution.

Institution	Number of publications	Frequency
Damascus	863	58%
Aleppo	349	23%
Latakia	132	9%
Hama	34	2%
Homs	31	2%
Tartous	62	4%
Daraa	19	1%
Total	1490	100%

Out of 1403 papers, 728 were carried out in partnership with external institutions.

**Fig. 4:**
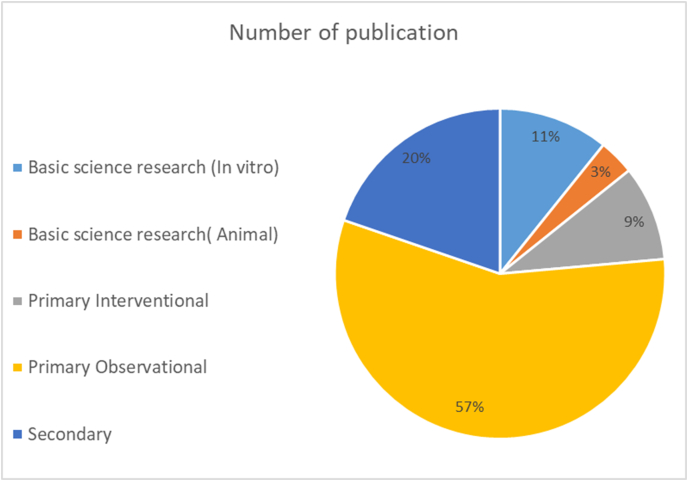
Pie chart illustrates the percentage of papers according to research type.

The prevailing study type was primary observational with 795 (56.7%) papers, followed by the secondary type with 277 (19.7%) papers. While primary interventional type counted 131 (9.3%) papers, basic scientific researches included 200 papers: 151(10.8%) of them were conducted in vitro and the other 49 (3.5%) researches were conducted on animals (Fig. 4).

**Fig. 5:**
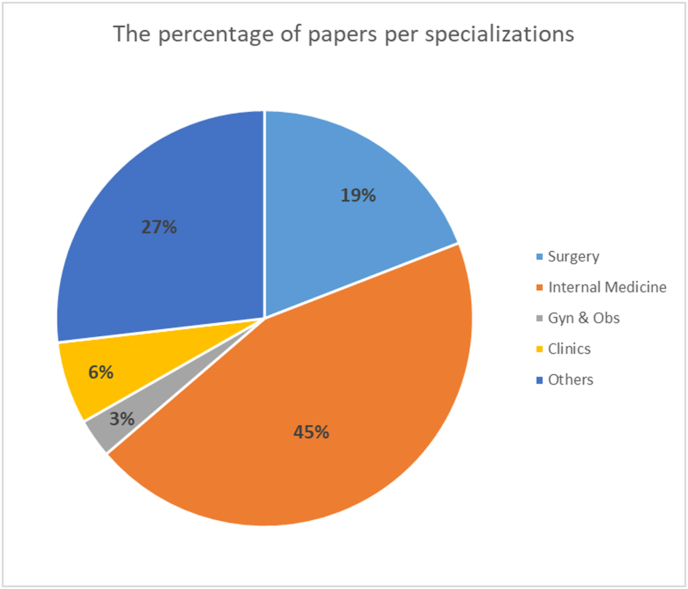
Pie chart illustrates the percentage of papers per specializations.

We have also classified the studies that were in medical field into their specialties (Fig. 5).

**Fig. 6:**
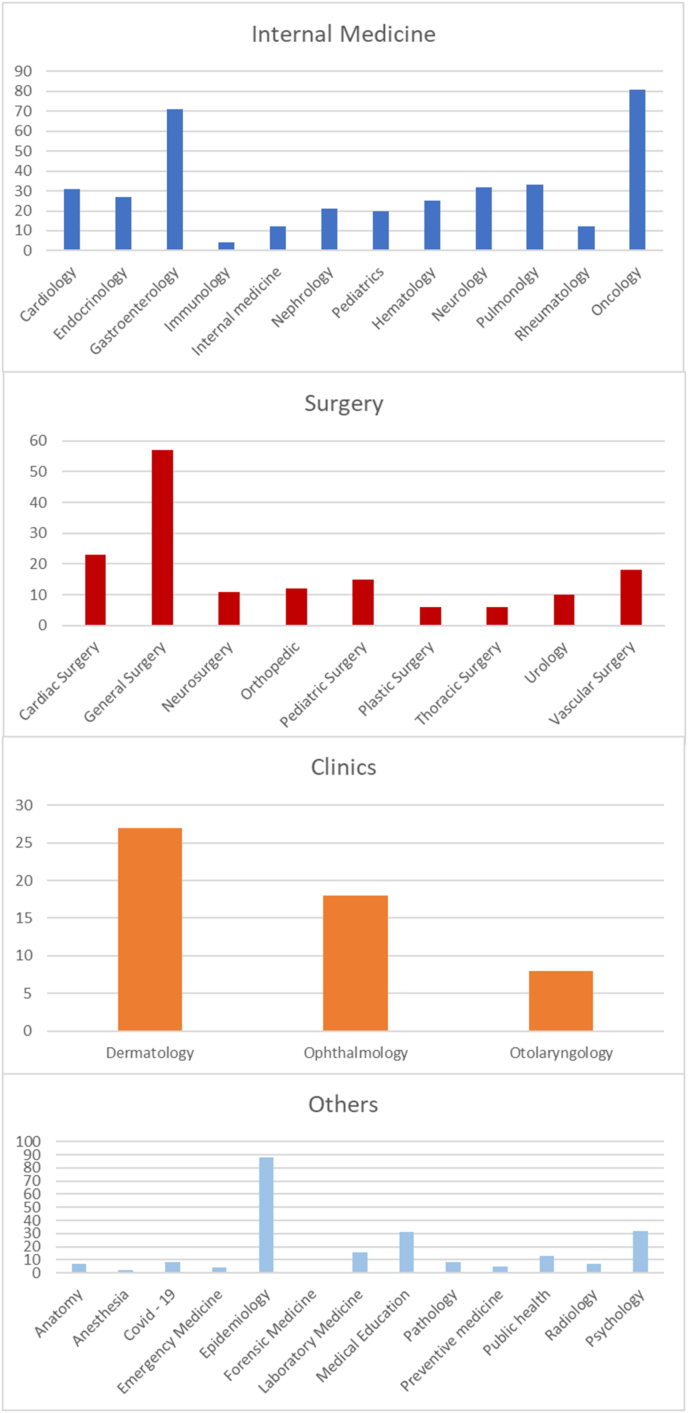
Number of papers in medicine field per specializations.

We found out that epidemiology achieved the highest percentage with 88 papers, oncology was in the second with 81 papers, and gastroenterology was in the third place with 71 papers. General surgery published the highest number of surgical papers with 57 papers; in contrast, both thoracic and plastic surgery had the lowest number of surgical papers, only 6 papers for each one. There were 32 publications in psychology, and only 8 papers were about COVID-19 pandemic. Forensic medicine had the lowest number of papers in general, with only one published paper. We also found out that 31 papers interested in general medical education (Fig. 6).

## 4 Discussion

This study provides a comprehensive review of the research productivity and health care practitioners’ situation in Syria during the last decade.

The Results showed paucity of publishing -in comparison with equivalent countries-, with ascending increase since 2011 and a slight peak in the last three years.

Syria, in total (in all fields), takes the 15th position of 22 Arab countries, and the 14th position out of 16 Middle Eastern countries, with 113 H-index and 8010 documents. While Jordan takes the 7th position of both Arab and Middle Eastern Countries in documents’ number with 176 H-index and 45884 documents, and Tunisia takes the third position of Arab Countries with 193 H-index and 94962 documents according to Scimago Journal & Country Rank [7]. (SCImago is a research group from the Consejo Superior de Investigaciones Científicas that is interested in Journals and country ranking.)

Paucity can be explained by many reasons such as: lack of mentors, being a low-income country, and having the current crisis. The crisis -which started in 2011- has been accompanied with economic and scientific sanctions, devastating social and demographic situations that constrained connecting with trials' participants, less financial resources, and immense stress on medical institutions [8,9]. At the same time, being a developing country caused the following: limited scientific funding, poor infrastructure such as: lack of electronic medical records, and immigration of minds [6,[10], [11], [12]].

More reasons can be mentioned: using Arabic as the official language in education -while English is the most widely used in research-, poor internet connection, limited participation and lack of training, important gap in medical knowledge in Syria, and insufficient motivation for medical students [5,9,13,14]. Moreover, an important reason is that some institutions refuse to deal with Syrian people in both educational and scientific fields because of the economic and scientific sanctions against Syria.

As for the peak, it was mainly caused by the increased awareness among physicians and medical students [15], which grew due to: enrolling in free online courses, adding scientific research to the curriculum of medical colleges, willing to gain external scholarships, and using social media to be acquainted with the latest publications and another online courses. Another prominent reason is the waiver of publication fee that is offered to researchers from low-income countries.

According to our data: Damascus affiliations reached the highest number of publications, because of having more experienced seniors and mentors, and more encouraging scientific atmosphere [6]. On the other hand, Aleppo and Latakia affiliations got the second and the third position, respectively; as scientific research teams have recently established there.

Medicine colleges published almost 828 papers, whereas dentistry and pharmacy colleges together published only 575 papers. This can be explained by the orientation of medical students toward evidence-based medicine (EBM) practice and continuous medical education, and updating educational curricula by adding EBM subject to the medical curricula.

The dominant proportion of these studies were observational (56.7%), which need less time, less funding and fewer experiments [16]. Secondary type took the second place with a majority of external collaborations, as a way to overcome the lack of professional expertise and weak scientific infrastructures.

The leading specialties were: epidemiology, oncology, and gastroenterology respectively. The priority of these three specialties is caused by having more active mentors, and many external contributions, especially by Tobacco Center affiliated researchers [17]. Psychiatry publications counted only 32 papers of 828, which is less than we expected as people have been suffering from war for years [18]. It can also be attributed to the decreased public awareness of psychological diseases, and the lack of psychiatrists. The recent crisis has presented important risk of chronic physical and mental illnesses which need to be studied [[19], [20], [21]].

### 4.1 Limitations and challenges

We did not include papers that their authors affiliated to institutions out of service due to Syrian war. We also did not do manual search for papers published locally in libraries. Moreover, included studies were evaluated depending on number only, neither citations nor quality of journals was considered.

### 4.2 Recommendations and solutions

As previously mentioned, there is an apparent difference in publication between governorates and faculties.

Alhamid et al. (2017) proposed solutions to overcome the obstacles in research productivity among medical students such as: holding research workshops, improving research skills by expert members, and adding new opportunities in school curriculum [15].

So, we recommend holding annual evidence based medical, dental, and pharmaceutical conferences to keep up with the latest updates, and to enhance internal collaborations to share the available expertise equally among governorates.

To increase the general scientific outcome, seniors and mentors should give students the chance to collaborate in their latest researches, as the recent studies have shown that the early young researchers are involved in publication, the more they achieve in future [22].

We advise Syrian students to improve their academic English language because it has a higher number of citations, readings and it is accessible to a larger audience [23,24]. Likewise, we advise them to work on secondary type papers such as Systematic Review, as they are considered a higher quality evidence and do not need much funding or effort. However, they also have to keep up with the latest publications of all types.

## 5 Conclusion

We noticed a peak in quantity of Syrian medical, dental and pharmaceutical publications in the last decade. Depending on our data, we expect this number of publications to increase in the next few years. Accordingly, we recommend enhancing research skills, paying more attention to the quality of researches, and holding research workshops and Evidence Based conferences to enhance the scientific endeavor.

## Funding

There are no funding sources.

### Ethical approval

There is no need.

### Sources of funding

There is no source of funding.

## Authors' contributions

MZBA: contributed to project administration, literature search, study design, data analysis, data interpretation, writing, reviewing, and editing. Both HS and RB: contributed to data analysis, data interpretation, literature search, writing, reviewing, and editing. AG: contributed to data analysis, data interpretation, software, study design, and creating figures. MNS: contributed to data analysis, data interpretation, and validation. LHK: contributed to data analysis, data interpretation, literature search, and writing. AG: contributed to supervision, data interpretation, planning, reviewing, and editing. All authors read and approved the final manuscript.

### Consent

This study does not have patients.

### Registration of Research Studies

1. Name of the registry: not applicable
2. Unique Identifying number or registration ID: not applicable
3. Hyperlink to your specific registration (must be publicly accessible and will be checked): not applicable

### Guarantor

Muhamad Zakaria Brimo Alsaman.

## Consent for publication

Not applicable.

## Availability of data and materials

All data generated or analyzed during this study are included in this article.

## Declaration of competing interest

The authors declare that they have no competing interests.
